# A Nationwide Analysis in France on Sex Difference and Outcomes Following Carotid Intervention in Asymptomatic Patients

**DOI:** 10.3390/jcm13196019

**Published:** 2024-10-09

**Authors:** Fabien Lareyre, Juliette Raffort, Riikka Tulamo, Gert J. de Borst, Christian-Alexander Behrendt, Christian Pradier, Roxane Fabre, Laurent Bailly

**Affiliations:** 1 Department of Vascular Surgery, Hospital of Antibes Juan-les-Pins, 06600 Antibes, France; 2 CNRS, Université Côte d’Azur, UMR7370, LP2M, 06107 Nice, France; 3 Fédération Hospitalo-Universitaire (FHU) Plan & Go, 06100 Nice, France; 4 Institute 3IA Côte d’Azur, Université Côte d’Azur, 06103 Nice, France; 5 Clinical Chemistry Laboratory, University Hospital of Nice, 06003 Nice, France; 6 Department of Vascular Surgery, Helsinki University Hospital and University of Helsinki, 00290 Helsinki, Finland; 7 Division of Vascular Surgery, Department of Surgery, University Medical Centre Utrecht, 3584 CX Utrecht, The Netherlands; 8 Asklepios Clinic Wandsbek, Asklepios Medical School, 22043 Hamburg, Germany; 9 Brandenburg Medical School Theodor Fontane, 16816 Neuruppin, Germany; 10 Public Health Department, University Hospital of Nice, Université Côte d’Azur, 06103 Nice, France; 11 Fédération Hospitalo-Universitaire INOVPAIN, University Hospital of Nice, Université Côte d’Azur, 06103 Nice, France; 12 Clinical Research Unit of the Côte d’Azur (UR2CA), Université Côte d’Azur, 06103 Nice, France

**Keywords:** sex differences, sex factors, outcomes, carotid, carotid endarterectomy, carotid artery stenting, national data, registry

## Abstract

**Objective:** The impact of sex on outcomes following carotid endarterectomy (CEA) and carotid artery stenting (CAS) is not fully elucidated. The aim of this study was to analyze the association between sex and outcomes of asymptomatic patients who underwent primary carotid interventions in France. **Methods:** This nationwide retrospective study was performed using the French National Health Insurance Information System and included asymptomatic patients who underwent primary carotid intervention over a 10-year period (1 January 2013 to 31 August 2023). Symptomatic patients and patients who had peri-operative neurologic events were excluded. The primary endpoints were the occurrence of death and stroke/transient ischaemic attack (TIA) at 30 days, 1 and 5 years after patients’ discharge. **Results:** In total, 115,879 patients were admitted for an index CEA (29.4% women) and 6500 for CAS (29.8% women). In the CEA group, no significant sex-related difference was observed for 30-day mortality; however, women had significantly lower 1-year and 5-year mortality rates compared to men (1.9% vs. 2.6%, *p* < 0.001 and 7.9% vs. 11.1%, *p* < 0.001). In the CAS group, women had lower 30-day, 1-year and 5-year mortality (0.6% vs. 1.0%, *p* = 0.040, 3.8% vs. 4.9%, *p* = 0.048, and 10.4% vs. 15.0%, *p* < 0.001). A multivariate analysis showed that sex was not associated with the risk of stroke/TIA and mortality at 30 days (OR 0.84 (95% CI 0.67–1.04) and 1.27 (95% CI 0.98–1.64)). Male sex was associated with a higher risk of 1-year and 5-year mortality (OR 1.24 (95% CI 1.13–1.36) and 1.25 (95% CI 1.18–1.31)), but a lower risk of stroke/TIA than female sex. **Conclusions:** No significant sex-related difference was observed at 30 days in patients being discharged alive and without peri-operative neurologic events. Male sex was associated with a higher risk of mortality but a lower risk of stroke/TIA at 1 and 5 years.

## 1. Introduction

Atherosclerotic carotid artery disease is associated with the risk of ischaemic stroke [1]. Carotid endarterectomy (CEA) has been proven to be a safe and effective treatment for reducing the long-term risk of disabling stroke. Carotid artery stenting (CAS) has also been proposed as a less invasive alternative to CEA [1,2,3]. Sex-related differences have been reported regarding the anatomical and pathophysiological features of the carotid artery. Women have smaller carotid artery diameters, and carotid plaque morphology differs from men [4,5]. A systematic review and meta-analysis reported that men have more frequently larger plaques, with more often calcifications, lipid-rich necrotic core, intraplaque hemorrhage or ulcerated plaque compared to women [6]. In addition, biological sex-related differences, including hormonal changes, have an impact on the vasculature, potentially causing sex-related differences in the risk of stroke and outcomes after carotid intervention [7,8].

A few randomized controlled trials (RCTs) analyzed the impact of sex on the outcomes of asymptomatic patients undergoing carotid revascularization. The Asymptomatic Carotid Stenosis Trial 1 (ACST-1) reported a similar benefit between men and women at 10 years after CEA [9,10]. Several observational studies aimed to address sex differences in the outcomes of patients following carotid intervention, with heterogeneous results. A systematic review and meta-analysis on the efficacy and safety of revascularization procedures in 332,344 symptomatic and asymptomatic patients found no sex-related differences regarding the rates of stroke or death, while the rate of re-stenosis at 1 year was significantly higher in women compared to men [11]. In a dataset of 8445 asymptomatic patients who underwent CAS, a significantly higher risk of in-hospital myocardial infarction was observed in women, but no sex difference was reported in the risk of post-procedural stroke [11]. A recent registry study led by VASCUNET, including over 200,000 carotid procedures (CEA and CAS) from 13 countries, reported no sex-related differences in the incidence of postoperative complications, including stroke, death and major cardiac events after adjusted analysis [12]. However, the VASCUNET study did not investigate long-term outcomes and included a limited number of patients from France.

While RCTs are considered high-quality evidence to support guideline development, the eligibility criteria used might not always reflect the patient population encountered in daily practice [12,13,14,15]. Women are often underrepresented in clinical trials, leading to low sex-related evidence. The impact of sex on outcomes following carotid intervention is, therefore, not yet completely elucidated, with heterogeneous results reported from current literature. Additional valuable insights can be retrieved from a single country’s national prospective registry with uniform reporting standards. The aim of this study was to analyze sex differences in the outcomes of asymptomatic patients who underwent primary carotid interventions, including CEA and CAS, in France over a ten-year retrospective period. The study population was restricted to asymptomatic patients who did not have peri-operative neurologic events during the first index stay.

## 2. Material and Methods

### 2.1. Study Population

This nationwide retrospective observational study was performed using longitudinal data collected from the French National Health Insurance Information System, which compiles standardized data on all patients admitted into all French public and private hospitals [16]. It is used for reimbursement by the Health National Insurance and, therefore, covers activities in all healthcare institutions in France. It compiles anonymous discharge reports completed and coded at the end of each hospital stay. As previously described, it provides exhaustive information on all surgical interventions performed in France, including vascular interventions [17,18]. In this database, variables are defined according to the World Health Organization (WHO) International Classification of Diseases (ICD-10) and the Common Classification of Medical Acts (CCAM). Data were extracted from over a ten-year retrospective period using these standardized classifications (Supplemental Table S1).

Inclusion criteria were asymptomatic patients (older than 18 years) admitted for primary carotid intervention in any public or private hospital between 1 January 2013 and 31 August 2023. They were identified in the database using the following methodology (Supplemental Figure). Patients who underwent carotid intervention, namely CEA and CAS, as defined by the corresponding CCAM codes, were searched (Supplemental Table S1). Then, exclusion criteria were applied to select asymptomatic patients who had primary carotid intervention. The exclusion criteria applied were patients younger than 18 years, patients who had a stroke or transient ischaemic attack (TIA) during index stay or in their past medical history, and patients who had previous CEA or CAS in their past medical history. Stroke and TIA were defined by the ICD-10 codes (I63, I64 and G45, respectively) (Supplemental Table S1). As the French National Health Insurance Information System did not provide coding systems, allowing symptomatic patients to be distinguished from patients who had peri-operative stroke/TIA at the first index stay, these patients were excluded from the analysis.

### 2.2. Data Collection, Endpoints and Follow-Up

Patient data and follow-up data were collected from the French National Health Insurance Information System, which identifies each patient with a unique alphanumerical anonymous identifier. Collected data included age (in years), sex (dichotomized) and comorbidities, including diabetes, dyslipidemia, arterial hypertension, smoking, alcohol consumption, obesity, congestive heart failure, ischaemic heart disease, chronic respiratory disease, severe chronic kidney disease, chronic liver disease, solid tumor, and Charlson Comorbidity Index [19]. Comorbidities were defined according to ICD-10 classification, and the codes used are fully described in Supplemental Table S1.

The primary endpoints were the occurrence of death and of stroke/TIA at 30 days, 1 year and 5 years during any re-hospitalization after the index stay. The follow-up of patients was evaluated starting from inpatient discharge to the date of the last re-hospitalization for any medical reason. Patients who did not have any re-hospitalization were thus excluded from the follow-up. Overall mortality was defined as any death after the index CEA or CAS. In the hospital, mortality was defined as deaths occurring during the index stay and total mortality was evaluated until the last follow-up of patients. 30-day, 1- and 5-year mortalities were evaluated from inpatient discharge; therefore, excluding patients who had died during the in-hospital stay. The occurrence of stroke/TIA was evaluated following the same methodology and started from inpatient discharge. Eligibility criteria excluded patients who had peri-operative stroke/TIA during the first index stay.

### 2.3. Ethical Approval

The protocol was approved by the Institutional Review Board of the University Hospital of Nice and by the French National Health Insurance Information System (Système National des Données de Santé, SNDS). The study was conducted in accordance with the French National Health Data Institute regulation (Institut National des Données de Santé, INDS). Informed consent was waived in accordance with the French National Health Data Institute regulation and guidelines on the use of routinely collected research data.

### 2.4. Statistical Analysis

Characteristics and outcomes of asymptomatic patients who underwent carotid intervention were expressed as means ± standard deviation (SD) or median with interquartile range for continuous data and as numbers with percentages for categorical data. A univariate analysis comparing characteristics and outcomes in men and women was run for CEA and CAS procedures separately. Group differences were analyzed using the Student *t*-test for quantitative variables and the Chi-2 test for categorical variables. A *p*-value less than 0.05 was considered statistically significant. Cumulative Kaplan–Meier estimates, and the log-logistic survival curves were analyzed, and it was shown that the proportional hazards assumption did not hold, hindering the validity of the Cox regression analysis. Therefore, logistic regression was applied to test whether sex was associated with mortality or stroke/TIA after a carotid procedure in asymptomatic patients at 30 days, 1 year and 5 years. Variables included in the models were selected on an a priori decision, and interactions between variables were checked. The selection of variables included in the multivariate analysis was based on current literature that identified the main factors that may impact mortality and stroke/TIA [1,2,3]. It included age, sex, type of procedures, and comorbidities encompassed in the Charlson Comorbidity Index. Effect sizes were expressed as odds ratios (OR) with a 95% confidence interval (CI) using the Wald method. The discriminative ability of the model was assessed using C-statistic, and the likelihood ratio was used to evaluate the relationship between variables and the predicted outcome. All statistical analyses were performed using SAS version 9.4 (SAS Institute, Cary, NC, USA).

## 3. Results

### 3.1. Baseline Characteristics

In total, 115,879 patients were admitted for an index CEA (29.4% women) and 6500 were admitted for an index CAS (29.8% women) (Table 1). Among patients who underwent CEA, significant differences were observed between women and men regarding clinical pre-operative characteristics. Women were significantly older (73.3 years vs. 71.6, *p* < 0.001) and had a higher prevalence of arterial hypertension and obesity than men (Table 1). However, women had a lower proportion of comorbidities, including diabetes, smoking habit and alcohol consumption, congestive heart failure, ischaemic heart disease, cardiac arrhythmia, chronic kidney disease, chronic liver disease, chronic pulmonary disease, or a solid tumor. In addition, they had a mean Charlson Comorbidity Index significantly lower (3.7 vs. 4.5, *p* < 0.001). In patients who underwent CAS, women were younger than men (69.4 years vs. 70.9, *p* < 0.001), and they had significantly fewer comorbidities (mean Charlson Comorbidity Index at 4.1 vs. 5.2, *p* < 0.001) than men.

### 3.2. Outcomes of Asymptomatic Patients Who Underwent Carotid Intervention in France

In the group of patients who underwent CEA, the total number of deceased patients was 17,234 deaths (14.9%) during a median follow-up of 2.6 (0.4–5.3) years (Table 2). During the follow-up, data were available for 79% (78,197/98,962) of patients at 1 year and for 54.9% (31,397/57,173) at 5 years. The 30 day and 1 year mortality were 280 (0.2%) and 2740 (2.4%), respectively. Women had significantly lower overall mortality rates compared to men (11.6% vs. 16.2%, *p* < 0.001). The 1-year and 5-year mortality were also significantly lower (1.9% vs. 2.6%, *p* < 0.001 and 7.9% vs. 11.1%, *p* <0.001, respectively). No significant difference was observed between men and women regarding the incidence of stroke/TIA during the follow-up in the univariate analysis. In patients who underwent CAS, the overall mortality was 1163 deaths (17.9%) for a median follow-up of 1.9 (0.2–4.6) years. Follow-up data were available for 76.4% (3986/5219) of patients at 1-year and for 50.6% (1409/2784) at 5-year. Overall mortality was significantly lower in women compared to men (13.8% vs. 19.6%, *p* < 0.001). Women had lower 30-day, 1-year and 5-year mortality rates compared to men (0.6% vs. 1.0%, *p* = 0.040, 3.8% vs. 4.9%, *p* = 0.048, and 10.4% vs. 15.0%, *p* < 0.001).

### 3.3. Factors Associated with TIA/Stroke and Mortality

The multivariate analysis of factors associated with 30-day outcomes showed that the type of surgical intervention was significantly associated with the risk of 30-day stroke/TIA and 30-day mortality, with odds ratio (OR) 2.86 (95% CI 2.16–3.77) and 2.46 (95% CI 1.76–3.43) for CAS as compared to CEA, respectively (Table 3). The Charlson Comorbidity Index was also associated with the risk of stroke/TIA and mortality (OR 1.13 (95% CI 1.10–1.16) and 1.10 (95% CI 1.07–1.14)) (Table 3). Sex was not associated with the risk of mortality at 30-day. The multivariate analysis at 1-year showed that CAS and the Charlson Comorbidity Index were significantly associated with stroke/TIA and mortality (Table 4). Male sex was significantly associated with the risk of 1-year mortality (OR 1.24 (95% CI 1.13–1.36) (Table 4).

The analysis at 5 years confirmed the association between the Charlson Comorbidity Index and the risk of stroke/TIA (OR 1.22 (95% CI 1.21–1.23)) and mortality (OR 1.32 (95% CI 1.31–1.33)), respectively) (Table 5). Male sex was associated with a higher risk of 5-year mortality than female sex (OR 1.25 (95% CI 1.18–1.31)) (Table 5). No significant association was observed at 5 years between CAS and the risk of mortality (OR 1.04 (95% CI 0.95–1.14) and the risk of stroke/TIA (OR 1.02 (95% CI 0.88–1.17) (Table 5).

Regarding the risk of stroke/TIA, no significant sex-related difference was observed at 30 days after the multivariate analysis (Table 3). However, men had a lower risk of TIA/stroke compared to women at 1-year and 5-year (OR = 0.89 (0.81–0.99) and 0.86 (0.80–0.93), respectively) (Table 4 and Table 5).

### 3.4. Discussion

This French nationwide analysis reported the outcomes of 122,379 asymptomatic patients who underwent primary carotid intervention, including 115,879 (94.7%) CEA and 6500 (5.3%) CAS. It aimed to identify sex differences on stroke/TIA and mortality during follow-up after patients’ discharge. Due to the unavailability of appropriate codes in the French National Health Insurance Information System, the analysis excluded patients who had peri-operative stroke/TIA during the first index stay. Due to this limitation, the reported numbers for stroke/TIA and deaths may remain smaller in comparison to the possibility of inclusion of these patients in the analyzed datasets. Despite this limitation, to the best of our knowledge, this is one of the largest reported national cohorts.

In the current study, women had significantly less comorbidities than men. However, no sex-related difference was identified for the risk of stroke/TIA and mortality occurring 30 days following discharge after the multivariate analysis and the adjustment on confounding factors. These results are in accordance with a recent analysis by the VASCUNET group, which reported 223,626 carotid artery procedures from 13 countries, of which 111,418 were on asymptomatic patients (48,737 CEA and 62,681 CAS). The study found no association between sex and 30-day stroke and mortality [12].

The multivariate analysis adjusted for age, type of procedure and comorbidities identified a significant association between male sex and the risk of 1 and 5-year mortality. Several studies aimed to address sex differences in the outcomes of patients undergoing carotid intervention [11,20,21,22,23,24,25,26,27,28,29,30,31,32]. Among them, a Swedish observational study involving 1033 patients who underwent CEA in two centers showed that men had a higher mortality rate within the 10 years after CEA compared to women [21]. Further, a long-term Vascular Quality Initiative (VQI) follow-up analysis of asymptomatic patients treated with CAS found higher all-cause mortality in male patients compared with their female counterparts (26.9 vs. 15.7%, *p* < 0.001) [22]. A recent meta-analysis on patients who underwent major vascular surgery also showed that female patients treated by CAS had lower all-cause mortality compared to men (RR 0.85; 95% CI 0.76–0.94) [25].

In the current study, no significant difference was observed regarding stroke/TIA rates between men and women after CEA or CAS at 30 days, but male sex was associated with a lower risk of stroke/TIA at 1 and 5 years in the multivariate analysis. However, these results should be interpreted with caution since the current study excluded patients who had peri-operative neurologic events. Another 10-year nationwide analysis across the United States, including 1242 688 carotid interventions, corroborates this finding, where asymptomatic men and women had a similar risk of stroke after both CEA (OR 0.95; 95% C 0.79–1.14) and CAS (OR 0.70; 95% CI 0.43–1.13) [20]. However, in the current study, CAS was associated with the risk of stroke/TIA and the risk of mortality at 30 days and at 1 year. This is in accordance with the VASCUNET study, which identified CAS vs. CEA as independent risk factors of 30-day stroke and mortality [12]. We found that the association between CAS and the risk of stroke and mortality was no longer significant after five years of follow-up. The Second Asymptomatic Carotid Surgery Trial (ACST-2) also showed that the outcome of CEA and CAS was similar, with no significant difference in the rate of fatal or disabling stroke at 5 years [33]. However, reports from the German nationwide insurance claims involving 22,637 carotid interventions and 15,005 asymptomatic patients showed that CAS was significantly associated with the composite endpoint of death or stroke after a 5-year follow-up, with a Hazard ratio of 1.27 (95% CI 1.15–1.40, *p* < 0.001) [34].

The current study involved 5.3% of patients who underwent CAS, which is in the range of the proportion of CAS patients in the previous registry studies by both the VQI and VASCUNET (0 and 26%) [35]. Also, the proportion of women (30%) in the current study was comparable to international practice, with a mean proportion of women of 37%, ranging from 12% in Finland to 41% in the United States [35]. Furthermore, the proportion of women did not differ between CEA and CAS, which is similar to what was observed in other countries [35].

While RCTs have suggested an increased risk in peri-operative outcomes in women, recent literature acknowledges that women have been underrepresented in clinical trials and thus, results from real-world data may differ [12,13,26]. The VASCUNET study investigated sex differences after carotid interventions on real-world data and did not identify significant differences in stroke and/or death within 30 days of carotid revascularization [12]. The recent European Society for Vascular Surgery (ESVS) clinical practice guidelines do not provide sex-specific recommendations regarding the indications or choice of carotid procedures [1]. Although further studies are required, this nationwide analysis can be useful to better understand sex differences in outcomes following carotid revascularization.

Biological mechanisms underlying sex differences in the outcomes after carotid intervention remain to be explored. Women have smaller carotid arteries than men and differences regarding plaque composition have been reported [4,6,36,37]. In addition, vascular ageing differs between sex and is impacted by hormonal changes [7]. Although further studies are required, differences regarding carotid anatomy, plaque composition and vascular ageing may have contributed to differences on the outcomes after carotid intervention.

This study presents several strengths and limitations. Data from the French National Health Insurance Information System enabled an analysis of a complete patient cohort, covering both private and public hospitals representative of the whole French population, and provided adequate statistical power to investigate differences between men and women. However, because the national database was initially designed for administrative and financial purposes, some procedural-related data or pharmaceutical treatments relevant to the research purposes were not all available. For the same reason, the severity and degree of carotid stenosis were not available either. In addition, the current coding system could identify only asymptomatic patients with certainty. Thus, symptomatic patients and patients who had a peri-procedural stroke during the first index stay were not included in the study. Finally, events occurring in the absence of hospitalization could not be recorded in the database. It may have contributed to lower mortality rates in the reported cohort.

Detailed information regarding outcomes, such as the severity of the stroke or the cause of death, could not be evaluated. The results of the study may depend on the reliability and the quality of the coding system used by the national French electronic health data. However, the database is evaluated and audited every year by experts from the national health system to ensure the comprehensiveness and quality of data. Due to the observational design of the study, random assignment could not be performed, but main confounding factors could be taken into account using multivariable regression models.

## 4. Conclusions

This nationwide analysis investigated the outcomes of asymptomatic patients who underwent carotid revascularization in France and were discharged alive and with no peri-operative neurologic events. No significant sex-related difference was observed regarding the occurrence of stroke/TIA and mortality 30 days after discharge. Male sex was significantly associated with the risk of 1-year and 5-year mortality and lower risk of 1-year and 5-year stroke/TIA risk than female sex after the multivariate analysis. This study emphasizes an interest in the reporting of real-world data obtained from a national database and vascular registries to complement results obtained from clinical trials, bringing evidence to build clinical practice guidelines.

## Figures and Tables

**Table 1:** Characteristics of 122,379 asymptomatic patients who underwent carotid endarterectomy (CEA) or carotid artery stenting (CAS) in France in 2013–2023.

**CEA**		Total *	Men *	Women *	***p* Value**
	Number of patients	115,879	81,845 (70.6)	34,034 (29.4)	
	Age—years	72.1 ± 9.2	71.6 ± 9.0	73.3 ± 9.5	<0.001
	Arterial hypertension	68,675 (59.3)	47,828 (58.4)	20,847 (61.3)	<0.001
	Dyslipidæmia	34,455 (29.7)	24,274 (29.7)	10,181 (29.9)	0.39
	Diabetes	37,341 (32.2)	27,523 (33.6)	9818 (28.9)	<0.001
	Anemia	2439 (2.1)	1677 (2.1)	762 (2.2)	0.040
	History of smoking	11,797 (10.2)	8934 (10.9)	2863 (8.4)	<0.001
	Alcohol consumption	2505 (2.2)	2172 (2.6)	333 (1.0)	<0.001
	Obesity	10,406 (9.0)	7224 (8.8)	3182 (9.4)	0.005
	Congestive heart failure	11,390 (9.3)	9035 (11.0)	2355 (6.9)	<0.001
	Ischaemic heart disease	4003 (3.5)	3131 (3.8)	872 (2.6)	<0.001
	Cardiac arythmia	14,768 (12.7)	11,243 (13.7)	3525 (10.4)	<0.001
	Chronic kidney disease	7047 (6.1)	5160 (6.3)	1887 (5.5)	<0.001
	Chronic liver disease	5329 (4.6)	4129 (5.0)	1200 (3.5)	<0.001
	Chronic pulmonary disease	9849 (8.5)	7290 (8.9)	2559 (7.5)	<0.001
	Solid tumor	1806 (1.6)	1457 (1.8)	349 (1.0)	<0.001
	Charlson Comorbidity Index	4.3 ± 3.0	4.5 ± 3.0	3.7 ± 2.7	<0.001
**CAS**	Number of patients	6500	4564 (70.2)	1936 (29.8)	
	Age—years	70.4 ± 11.5	70.9 ± 10.0	69.4 ± 14.5	<0.001
	Arterial hypertension	3437 (52.9)	2421 (53.1)	1016 (52.5)	0.68
	Dyslipidæmia	2101 (32.3)	1463 (32.1)	638 (33.0)	0.48
	Diabetes	1846 (28.4)	1374 (30.1)	472 (24.4)	<0.001
	Anemia	134 (2.1)	84 (1.8)	50 (2.6)	0.054
	History of smoking	784 (12.1)	582 (12.8)	202 (10.4)	0.009
	Alcohol consumption	128 (2.0)	112 (2.5)	16 (0.8)	<0.001
	Obesity	1192 (18.3)	793 (17.4)	399 (20.6)	0.002
	Congestive heart failure	605 (9.3)	458 (10.0)	147 (7.6)	0.002
	Ischaemic heart disease	396 (6.1)	280 (6.1)	116 (6.0)	0.83
	Cardiac arythmia	777 (12.0)	597 (13.1)	180 (9.3)	<0.001
	Chronic kidney disease	739 (11.4)	519 (11.4)	220 (11.4)	0.99
	Chronic liver disease	325 (5.0)	253 (5.5)	72 (3.7)	0.002
	Chronic pulmonary disease	544 (8.4)	402 (8.8)	142 (7.3)	0.050
	Solid tumor	242 (3.7)	201 (4.4)	41 (2.1)	<0.001
	Charlson Comorbidity Index	4.9 ± 3.1	5.2 ± 3.1	4.1 ± 2.9	<0.001

Data are presented as *n* (%) or as mean ± standard deviation. * The percentages are calculated per total number of patients in the CEA and CAS groups.

**Table 2:** Outcomes of 122,379 asymptomatic patients who underwent carotid endarterectomy (CEA) or carotid artery stenting (CAS) in France in 2013–2023.

**CEA**		**Total**	**Men**	**Women**	***p* Value**
	In-hospital lenght of stay—days	4.0 (3.0–5.0)	4.0 (3.0–5.0)	4.0 (3.0–5.0)	0.27
	Overall mortality	17,234/115,879 (14.9) *	13,274/81,845 (16.2) *	3960/34,034 (11.6) *	<0.001
	In-hospital surgical mortality	242/115,879 (0.2) *	176/81,845 (0.2) *	66/34,034 (0.2) *	0.47
	30-day mortality	280/115,637 (0.2) †	208/81,669 (0.2) †	72/33,968 (0.2) †	0.18
	1-year mortality	2740/115,637 (2.4) †	2105/81,669 (2.6) †	635/33,968 (1.9) †	<0.001
	5-year mortality	11,765/115,637 (10.2) †	9085/81,669 (11.1) †	2680/33,968 (7.9) †	<0.001
	Total stroke/TIA	7062/115,879 (6.1) *	4978/81,845 (6.1) *	2084/34,034 (6.1) *	0.79
	30-day stroke/TIA	345/115,637 (0.3) †	238/81,669 (0.3) †	107/33,968 (0.3) †	0.50
	1-year stroke/TIA	1961/115,637 (1.7) †	1382/81,669 (1.7) †	579/33,968 (1.7) †	0.88
	5-year stroke/TIA	5533/115,637 (4.8) †	3909/81,669 (4.8) †	1624/33,968 (4.8) †	0.97
	Composite 30-day death+ stroke/TIA	779/115,637 (0.7) †	562/81,669 (0.7) †	217/33,968 (0.6) †	0.35
	Follow-up—years	2.6 (0.4–5.3)	2.7 (0.5–5.4)	2.3 (0.2–5.1)	<0.001
	Follow-up over 1 year	78,197/98,962 (79.0) ‡	56,130/70,024 (80.2) ‡	22,067/28,938 (76.3) ‡	<0.001
	Follow-up over 5 years	31,397/57,173 (54.9) ‡	22,764/40,622 (56.0) ‡	8633/16,551 (52.2) ‡	<0.001
**CAS**	In-hospital length of stay—days	3.0 (2.0–3.0)	3.0 (2.0–3.0)	3.0 (2.0–4.0)	0.27
	Overall mortality	1163/6500 (17.9) *	895/4564 (19.6) *	268/1936 (13.8) *	<0.001
	In-hospital surgical mortality	18/6500 (0.3) *	13/4564 (0.3) *	5/1936 (0.3) *	0.85
	30-day mortality	40/6482 (0.9) †	34/4551 (1.0) †	6/1931 (0.6) †	0.040
	1-year mortality	296/6482 (4.6) †	223/4551 (4.9) †	73/1931 (3.8) †	0.048
	5-year mortality	882/6482 (13.6) †	682/4551 (15.0) †	200/1931 (10.4) †	<0.001
	Total stroke/TIA	502/6500 (7.7) *	364/4564 (8.0) *	138/1936 (7.1) *	0.24
	30-day stroke/TIA	59/6482 (0.9) †	41/4551 (0.9) †	18/1931 (0.9) †	0.90
	1-year stroke/TIA	200/6482 (3.1) †	147/4551 (3.2) †	53/1931 (2.7) †	0.30
	5-year stroke/TIA	424/6482 (6.5) †	312/4551(6.8) †	112/1931 (5.8) †	0.12
	Composite 30-day death+ stroke/TIA	102/6482 (1.6) †	77/4551 (1.7) †	25/1931 (1.3) †	0.24
	Follow-up—years	1.9 (0.2–4.6)	2.1 (0.3–4.7)	1.6 (0.1–4.3)	<0.001
	Follow-up over 1 year	3986/5219 (76.4) ‡	2888/3697 (78.1) ‡	1098/1522 (72.1) ‡	<0.001
	Follow-up over 5 years	1409/2784 (50.6) ‡	1024/2014 (50.8) ‡	385/770 (50.0) ‡	0.72

Data are presented as *n* (%) or as median with interquartile range. TIA = transient ischaemic attack. * Cumulative data reported per total number of patients at index stay. † Cumulative data reported per total number of patients starting from discharge. ‡ Proportion of patients for whom follow-up data were available reported to the total theorical number of patients at the time point given inclusion period.

**Table 3:** Factors associated with 30-day stroke/transient ischaemic attack (TIA) and 30-day mortality after multivariate analysis in asymptomatic patients who underwent carotid endarterectomy (CEA) or carotid artery stenting (CAS) in France in 2013–2023.

	30-Day Stroke/TIA			30-Day Mortality		
	Adjusted OR	95% CI	*p* Value	Adjusted OR	95% CI	*p* Value
CAS (ref. CEA)	2.86	2.16–3.77	<0.01	2.46	1.76–3.43	<0.01
Charlson Comorbidity Index	1.13	1.10–1.16	<0.01	1.10	1.07–1.14	<0.01
Male sex (ref. female sex)	0.84	0.67–1.04	0.10	1.27	0.98–1.64	0.07
Age	1.01	0.99–1.02	0.06	1.04	1.03–1.06	<0.01

CI = confidence interval; OR = odds ratio; Ref = reference.

**Table 4:** Factors associated with 1-year stroke/transient ischaemic attack (TIA) and 1-year mortality after multivariate analysis in asymptomatic patients who underwent carotid endarterectomy (CEA) or carotid artery stenting (CAS) in France in 2013–2023.

	1-Year Stroke/TIA			1-Year Mortality		
	Adjusted OR	95% CI	*p* Value	Adjusted OR	95% CI	*p* Value
CAS (ref. CEA)	1.42	1.19–1.70	<0.01	1.73	1.51–1.98	<0.01
Charlson Comorbidity Index	1.20	1.18–1.22	<0.01	1.20	1.19–1.21	<0.01
Age	1.02	1.02–1.03	<0.01	1.04	1.03–1.04	<0.01
Male sex (ref. female sex)	0.89	0.81–0.99	0.03	1.24	1.13–1.36	<0.01

CI = confidence interval; OR = odds ratio; Ref = reference.

**Table 5:** Factors associated with 5-year stroke/transient ischaemic attack (TIA) and 5-year mortality after multivariate analysis in asymptomatic patients who underwent carotid endarterectomy (CEA) or carotid artery stenting (CAS) in France in 2013–2023.

	5-Year Stroke/TIA			5-Year Mortality		
	Adjusted OR	95% CI	*p* Value	Adjusted OR	95% CI	*p* Value
CAS (ref. CEA)	1.02	0.88–1.17	0.83	1.04	0.95–1.14	0.40
Charlson Comorbidity Index	1.22	1.21–1.23	<0.01	1.32	1.31–1.33	<0.01
Age	1.02	1.02–1.02	<0.01	1.04	1.04–1.05	<0.01
Male sex (ref. female sex)	0.86	0.80–0.93	<0.01	1.25	1.18–1.31	<0.01

CI = confidence interval; OR = odds ratio; Ref = reference.

## Data Availability

The datasets presented in this article are not readily available according to French National regulation. Requests to access the datasets should be directed to Système National des Données de Santé.

## Supplementary Materials

The following supporting information can be downloaded at: https://www.mdpi.com/article/10.3390/jcm13196019/s1, Table S1: Definition of study variables with classification and codes used CCAM = Common Classification of Medical Acts; ICD-10 = International Classification of Diseases. Figure S1: Please also add the Supplemental figure: Methodology used to identify asymptomatic patients (older than 18 years) admitted for primary carotid intervention (CEA or CAS) in any public or private hospital in France between 1 January 2013, and 31 August 2023. CAS: Carotid Artery Stenting. CCAM: Common Classification of Medical Acts. CEA: Carotid endarterectomy. ICD-10: International Classification of Diseases.
