# Supplementary material for: A Nationwide Analysis in France on Sex Difference and Outcomes Following Carotid Intervention in Asymptomatic Patients
Source: J Clin Med. 2024 Oct 9;13(19):6019. doi: 10.3390/jcm13196019 (PMC11477587; doi:10.3390/jcm13196019)
Supplement: Supplementary file 1 [file jcm-13-06019-s001.zip › jcm-3220296-supplementary.pdf]

Variables	Classification and codes
**Stroke**	ICD-10 : cerebral infarction(I63), stroke not specified as haemorrhage or infarction (I64)
**Transient ischaemic attack**	ICD-10: transient cerebral ischaemic attacks and related syndrome (G45)
**Carotid endarterectomy**	CCAM: carotid endarterectomy (EBFA012 , EBFA006, EBFA016, EBFA002, EBFA008, EBFA015)
**Carotid stenting**	CCAM: carotid stenting (EBAF001, EBAF003, EBAF009, EBAF011)
**Diabetes**	ICD-10 : type 1 diabetes mellitus (E10.0- E10.9), type 2 diabetes mellitus (E11.0- E11.9), malnutrition-related diabetes mellitus (E12.0- E12.9), other specified diabetes mellitus (E13.0-3E13.9), unspecified diabetes mellitus (E14.0- E14.9)
**Dyslipidemia**	ICD-10 : disorder of lipoprotien metabolism and other lipidaemias (E78)
**Arterial hypertension**	ICD-10 : hypertensive diseases (I10- I13, I15)
**Smoking**	ICD-10 : mental and behavioural disorders due to use of tobacco (F17)
**Alcohol consumption**	ICD-10 : mental and behavioural disorders due to use of alcohol (F10), niacin deficiency (E52), alcoholic polyneuropathy (G62.1), alcoholic cardiomyopathy (I42.6), alcoholic gastritis (K29.2), alcoholic fatty liver (K70.0), alcoholic cirrhosis (K70.3), alcoholic liver disease unspecified (K70.9), ethanol toxic effect (T51), alcohol rehabilitation (Z50.2), alcohol abuse counselling and surveillance (Z71.4), alcohol use (Z72.1)
**Obesity**	ICD-10 : obesity (E66)
**Congestive heart failure**	ICD-10 : hypertensive heart disease with congestive heart failure (I11.0), hypertensive heart and renal disease with congestive heart failure (I13.0), hypertensive heart and renal disease with both (congestive) heart failure and renal failure (I13.2), rheumatic heart disease unspecified (I09.9), cardiomyopathy congestive (I42.0), other cardiomyopathy (I42.5 - I42.9), cardiomyopathy in diseases classified elsewhere (I43.x), heart failure (I50.x), cardiovascular disorders originating in the perinatal period (P29.0)
**Ischeamic heart disease**	ICD-10 : ischaemic heart disease (I20), acute myocardial infarction (I21), subsequent myocardial infarction (I22), other acute ischaemic heart disease (I24), chronic ischaemic heart disease (I25)
**Cardiac arythmia**	ICD-10: atrioventricular and left bundle-branch block (I44.1- I44.3), pre-excitation syndrome (I45.6), conduction disorder unspecified (I45.9), paroxysmal tachycardia (I47.x), atrial fibrillation and flutter (I48.x), other cardiac arrhytmias (I49.x), tachycardia unspecified (R00.0), bradycardia unspecified (R00.1), other unspecified abnormalities of heart beat (R00.8), mechanical complication of cardiac electronic device (T82.1), adjustement and management of cardiac device (Z45.0), presence of electronic cardiac device (Z95.0)
**Chronic respiratory disease**	ICD-10 : chronic lower respiratory disease (J40 - J47), lung disease due to external agents (J60 - J67), chronic respiratory conditions due to chemicals (J68.4), chronic and other pulmonary manifestations due to radiation (J70), other specified pulmonary heart diseases (I27.8), pulmonary

	heart disease (I27.9),
**Chronic kidney disease**	ICD-10 : hypertensive renal disease with renal failure (I12.0), hypertensive heart and renal disease (I13), chronic kidney disease (N18), unspecified kidney failure (N19), disorders resulting from impaired renal tubular function (N25), care involving dialysis (Z49)
**Chronic liver disease**	ICD-10 : chronic viral hepatitis (B18), alcoholic liver disease (K70), toxic liver disease (K71.1, K71.3 - K71.5, K71.7), hepatic failure (K72), chronic hepatitis (K73), fibrosis and cirrhosis of liver (K74), other diseases of liver (K76.0, K76.2 - K76.9), liver transplant status (Z94.4), oesophageal varices (I85), gastric varices (I86.4)
**Solid tumor**	ICD-10 : malignant neoplasms (C00- C26, C30 - C34, C37- C41, C43, C45 - C58., C60 - C76, C97)
**Anaemia**	ICD-10 : iron deficiency anaemia secondary to blood loss (D50.0), other iron deficiency anaemia (D50.8), iron deficiency anaemia unspecified (D50.9), vitamin B12, folate and other nutritional anaemias (D51-D53)

Supplemental Table S1: Definition of study variables with classification and codes used

CCAM = Common Classification of Medical Acts; ICD-10 = International Classification of Diseases

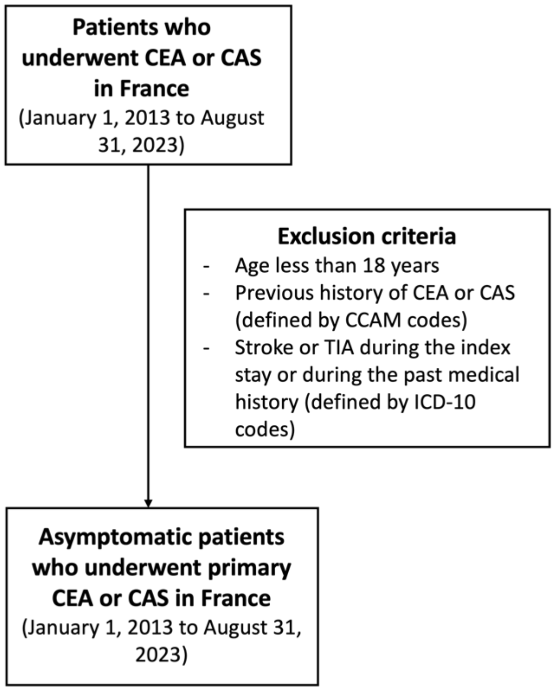

Supplemental Figure: Methodology used to identify asymptomatic patients (older than 18 years) admitted for primary carotid intervention (CEA or CAS) in any public or private hospital in France between January 1, 2013, and August 31, 2023.

CAS: Carotid Artery Stenting

CCAM: Common Classification of Medical Acts

CEA: Carotid endarterectomy

ICD-10: International Classification of Diseases
